# Linear scaling relationships and volcano plots in homogeneous catalysis – revisiting the Suzuki reaction†
†Electronic supplementary information (ESI) available: Detailed derivation of the linear scaling relationships and construction of the volcano plots as well as comparisons of computed values using PBE0-dDsC and M06 functionals is included. See DOI: 10.1039/c5sc02910d



**DOI:** 10.1039/c5sc02910d

**Published:** 2015-09-02

**Authors:** Michael Busch, Matthew D. Wodrich, Clémence Corminboeuf

**Affiliations:** a Laboratory for Computational Molecular Design , Institute of Chemical Sciences and Engineering , Ecole Polytechnique Fédérale de Lausanne (EPFL) , CH-1015 Lausanne , Switzerland . Email: clemence.corminboeuf@epfl.ch

## Abstract

Linear free energy scaling relationships and volcano plots are common tools used to identify potential heterogeneous catalysts for myriad applications. Despite the striking simplicity and predictive power of volcano plots, they remain unknown in homogeneous catalysis. Here, we construct volcano plots to analyze a prototypical reaction from homogeneous catalysis, the Suzuki cross-coupling of olefins. Volcano plots succeed both in discriminating amongst different catalysts and reproducing experimentally known trends, which serves as validation of the model for this proof-of-principle example. These findings indicate that the combination of linear scaling relationships and volcano plots could serve as a valuable methodology for identifying homogeneous catalysts possessing a desired activity through *a priori* computational screening.

## Abstract 2

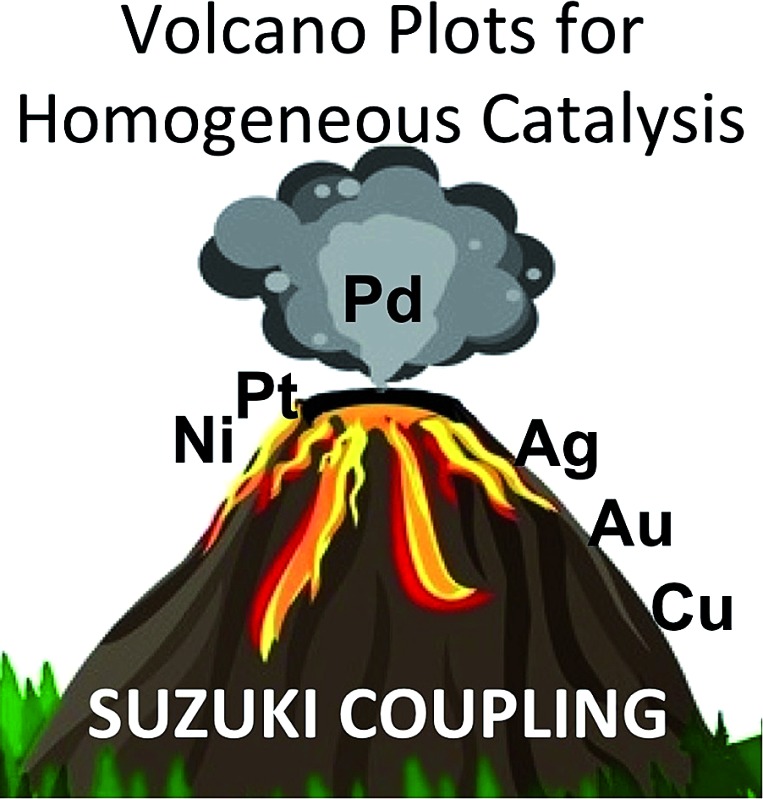
Volcano plots, commonly used to identify attractive heterogeneous catalysts are applied, for the first time, to a prototypical homogeneous system.

## Introduction

Nobel laureate Paul Sabatier conceived of an “ideal catalyst” in which interactions with a substrate are tuned to be neither too weak nor too strong.^1^ This phenomenon, commonly known as Sabatier's principle, has been cast into an intuitive tool, volcano plots, which pictorially characterize catalytic activity with respect to catalyst/intermediate interactions.^2,3^ The predictive power of these concepts has rendered them indispensible in modern heterogeneous catalysis and electrochemistry.^4–6^ Volcano plots contain a minimum of two slopes, meeting in a top. The volcano shape aids comparison of the thermodynamics between different catalysts, thereby facilitating identification of “good” candidates. Thermodynamically optimal candidates, those fulfilling Sabatier's principle, appear near the highest point of the volcano. The volcano slopes delineate situations in which the catalyst/substrate interaction is either too strong (left slope) or too weak (right slope). In computational chemistry, volcano plots are often constructed from linear free energy scaling relationships,^7^ which indicate that the relative stability of intermediates are dependent on one another.^8,9^

Given their ability to identify attractive catalysts as well as their conceptual simplicity, the idea of importing volcano plots from the heterogeneous community to the realm of homogeneous catalysis is wholly attractive. Indeed, many of the underlying principles of volcanoes, namely linear scaling relationships, are longstanding concepts associated with physical organic chemistry (*e.g.*, Hammett equation,^10^ Bell–Evans–Polyani principle^11,12^) and homogeneous catalysis (*e.g.*, Brønsted catalysis equation^13^) and are routinely used today in both the heterogeneous^14^ and homogeneous^15^ communities. Despite this, to the best of our knowledge, volcano plots for homogeneous systems have only been proposed in a hypothetical sense,^16^ but never brought into concrete existence. Here, we combine linear free energy scaling relationships and volcano plots to re-examine a prototypical and well-studied reaction from homogeneous catalysis, the Suzuki cross-coupling^17–19^ of olefins (eqn (1)). This reaction was chosen in order to establish the viability of volcano plots as a tool for use in homogeneous catalysis. Validation requires determining the ability of volcanoes to reproduce experimentally determined data and trends for a restricted set of catalysts on a well-studied system. For this purpose, Suzuki coupling seems particularly appropriate, given the considerable amount of knowledge and understanding gained during the decades since its introduction. We stress that the primary objective of this work is not to predict new catalysts for the Suzuki coupling of olefins, but rather to definitively establish that volcano plots are capable of identifying thermodynamically attractive catalysts for homogeneous reactions. Only after this key objective has been unambiguously established can studies be extended to a broader scope of catalysts and other homogeneous reactions.1
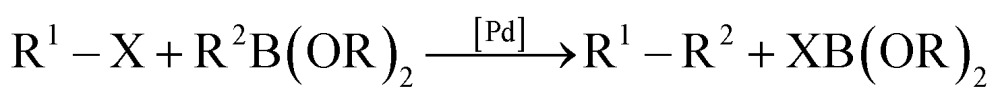



**Fig. 1:**
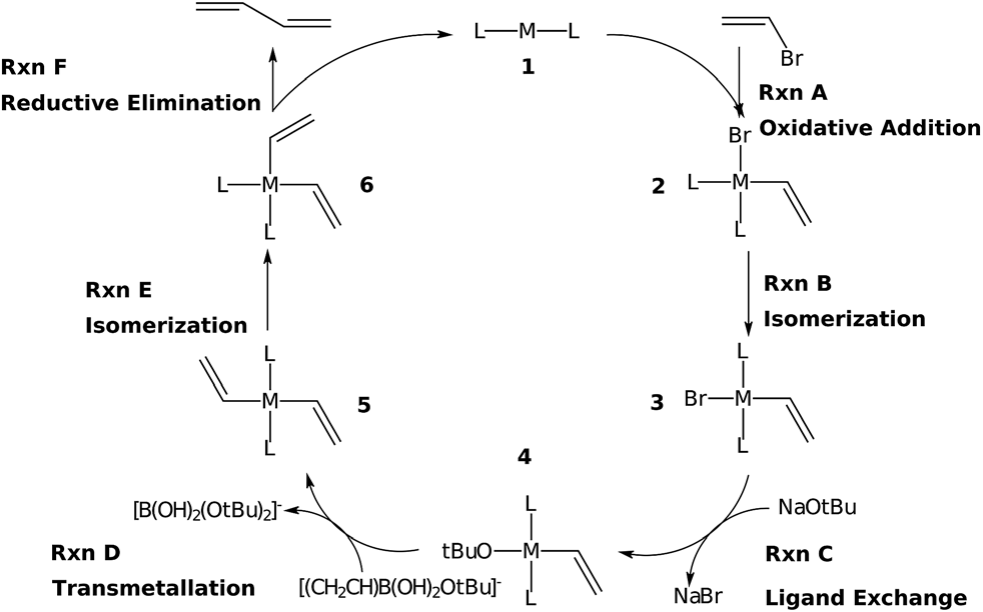
Reaction mechanism for the Suzuki cross-coupling of olefins.

The Suzuki reaction involves the coupling of an aryl or vinyl halogenide (R^1^–X) with an organoborate [R^2^B(OR)_2_] to form R^1^–R^2^ using a Pd catalyst (eqn (1)).^17–19^ The now well-established reaction mechanism^20–24^ proceeds as depicted in Fig. 1 with oxidative addition (Rxn **A**), *cis*/*trans* isomerisation (Rxn **B**), ligand exchange (Rxn **C**), transmetallation (Rxn **D**), *trans*/*cis* isomerisation (Rxn **E**), and reductive elimination (Rxn **F**) steps. During this cycle the catalyst proceeds through a series of five 16 electron square planar intermediates (**2–6**), the relative stabilities of which will become the basis of the linear scaling relationships (*vide infra*). While the reaction is known to proceed through these particular intermediates detailed knowledge of how the specific transitions occur between these intermediates is not necessary for creating insightful volcano plots.

Here, the manner in which different metal/ligand combinations influence the thermodynamics of the Suzuki reaction are probed using density functional theory computations. Because the goal of this work is establishing that the concepts of linear scaling relationships and volcano plots are as valuable for homogeneous systems as they are for predicting heterogeneous catalysts, we focus on simpler illustrative systems that demonstrate and reinforce these points. Note that several of these systems are expected to perform poorly, while others are expected to perform well. Taken together, these two limiting cases should effectively validate the power of volcano plots. Six metals (Ni, Pd, Pt, Cu, Ag, and Au) were combined with six ligand sets [CO (x2), NH_3_ (x2), PMe_3_ (x2), acetone (x2), an N-heterocyclic carbene (x2), and a mixed PMe_3_/NH_3_ system], the combinations of which produced 36 potential catalysts. To align with the chemistry of the Suzuki reaction, the oxidation states of the catalysts were adjusted to comply with the known 14e^–^/16e^–^ nature of the complexes. Of the 36 systems evaluated, 27 had stationary points for all catalytic cycle intermediates, while five had stationary points for only some intermediates. All of these species were used to establish linear scaling relationships.

## Computational details

Vinylbromide (H_2_CCHBr) and [H_2_CCH(O^*t*^Bu)(OH)_2_]^–^ were used as coupling partners and NaO^*t*^Bu and NaBr for ligand exchange. Geometries of all species were obtained by optimizations using the M06 (ref. 25 and 26) density functional along with the def2-SVP^27^ basis set in implicit THF solvent (SMD^28^ solvation model) with the “Ultrafine” integration grid in Gaussian 09.^29^ Refined energies were obtained by single point energy computations using a density-dependent dispersion correction^30–33^ (-dDsC) coupled with the PBE0 (ref. 34 and 35) functional along with the triple-ζ Slater type orbital basis set (TZ2P) in ADF.^36,37^ Final solvation corrections (also in THF) were determined using COSMO-RS,^38^ as implemented in ADF. Reported free energies include unscaled free energy corrections from M06/def2-SVP computations. Note that the combination of the -dDsC density dependent dispersion correction with COSMO-RS, as well as other solvation models, has been successfully used in numerous applications of catalysis with metal centres.^39–43^ The validity of the computational methodology was further confirmed *via* favourable comparisons with computations using the M06 functional combined with the SMD solvation model (*e.g.*, the same catalysts were identified as the most attractive thermodynamic candidates independent of functional choice, see ESI†). Selected species were also computed using alternate spin states, which revealed that the closed-shell singlet represented the ground state in all cases.

While we used static DFT computations as a tool to illustrate the ability of volcano plots to reproduce experimentally known trends from homogeneous catalysis, in principle, any number of computational or experimental techniques could also be employed. Since we tended to choose small, non-flexible ligands for our catalysts static DFT computations are appropriate. It could be foreseen, however, that larger, more bulky ligands residing on a catalyst (as often employed in experimental settings) might introduce problems arising from describing the free energy of a Boltzmann like conformer distribution using a single structure. In such a case, obtaining free energies from MD simulations, which specifically include the influences of conformational entropy,^44^ would be a fitting alternative.

## Results and discussion

### Free energy plots

**Fig. 2:**
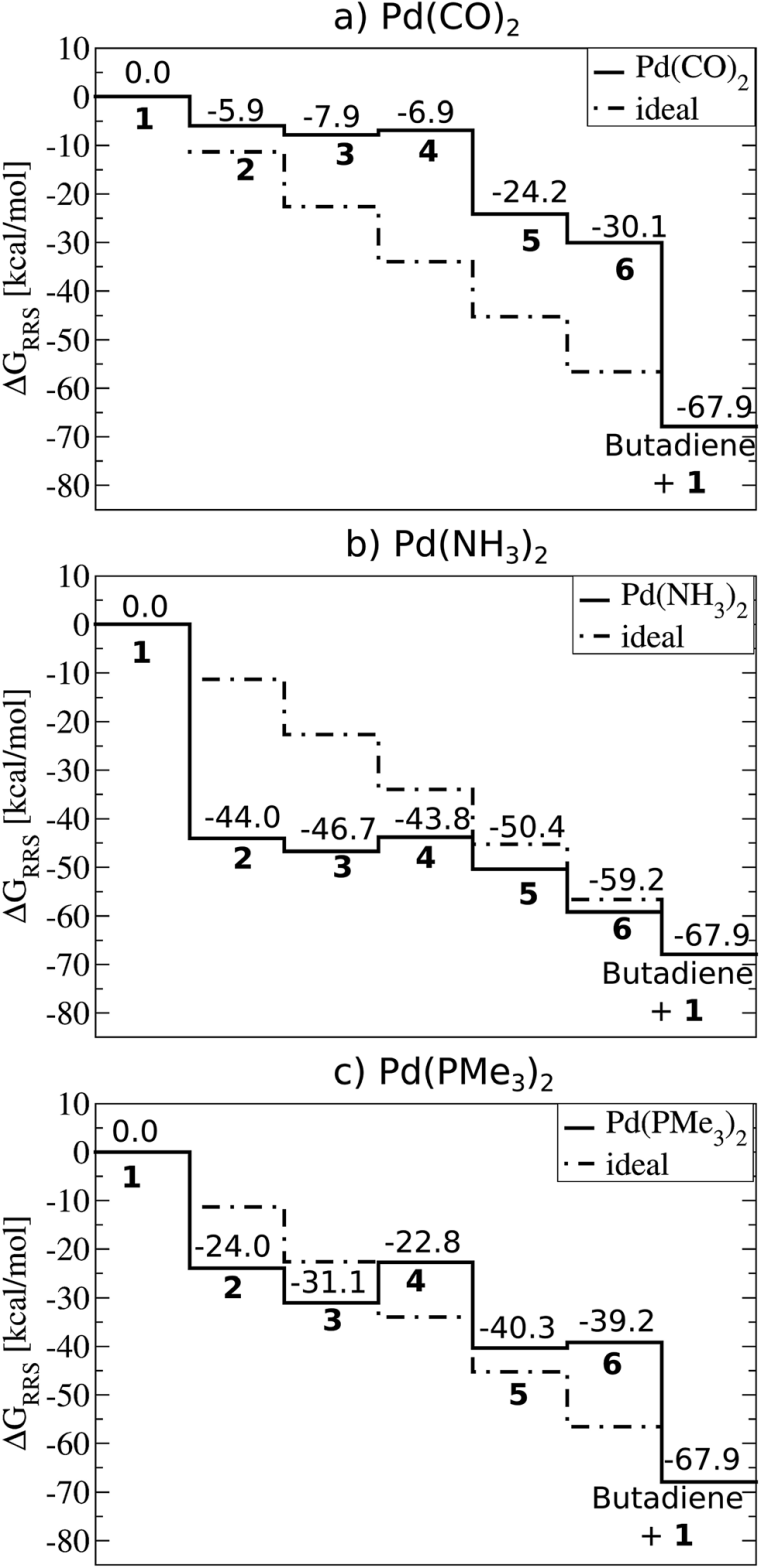
Free energy plots (PBE0-dDsC/TZ2P//M06/def2-SVP, COSMO-RS solvation) relative to the resting state (Δ*G*_RRS_, defined by eqn (2)–(6)) for selected catalysts: (a) Pd(CO)_2_, (b) Pd(NH_3_)_2_, (c) Pd(PMe_3_)_2_. Moving between different species (**1–6**) corresponds to completing the corresponding reactions (**A–F**) given in Fig. 1.

Fig. 2 depicts computed free energy diagrams describing the reaction energetics of Suzuki coupling for three exemplary catalytic systems. From a thermodynamic perspective, an ideal catalyst would proceed through the Fig. 1 catalytic cycle *via* a series of equally exergonic reaction steps, thereby making each intermediary reaction equally facile, assuming thermodynamic control. Such a situation, on average, would minimize the reverse reaction rate for each individual step and drive the system in a consistent manner toward the products (dotted lines, Fig. 2). Of course, this situation seldom occurs; for the particular case of Suzuki coupling no “ideal catalyst” is observed. Instead, the behaviour of different catalysts deviates, to a greater or lesser degree, depending on their specific properties. The largest deviations from the behaviour of a hypothetical ideal catalyst (dotted lines, Fig. 2) tend to appear in the oxidative addition and reductive elimination intermediary steps. While other metal/ligand combinations exhibit more extreme behaviour (see ESI† for more dramatic cases) the trends amongst several Pd based catalysts more typically associated with Suzuki coupling are quite illustrative.

For example, Pd(CO)_2_ is a representative case of a catalyst where the intermediate species are destabilized. This situation is defined by intermediates lying above the “ideal” line, such as in the Fig. 2a plot. Catalysts with destabilized intermediates have oxidative addition steps that are generally less exergonic then for the hypothetical ideal catalyst. For Pd(CO)_2_, the value of oxidative addition is only –5.9 kcal mol^–1^, considerably less than the –11.3 kcal mol^–1^ of the hypothetical ideal catalyst. The contrasting case, where intermediates are over stabilized, is seen in Pd(NH_3_)_2_ (Fig. 2b). Here oxidative addition is strongly exergonic, with a value of –44.0 kcal mol^–1^, greatly exceeding the ideal –11.3 kcal mol^–1^ of the hypothetical ideal catalyst. This strongly exergonic intermediary reaction causes most intermediates to fall below the “ideal” line. Because the free energy to complete one catalytic cycle is fixed (–67.9 kcal mol^–1^ for the Suzuki coupling studied here) an oxidative addition step that is either overly or underly exergonic must be balanced elsewhere in the catalytic cycle. This energetic compensation is seen in the cycle's ultimate step, reductive elimination. Systems with destabilized intermediates (*e.g.*, weakly exergonic oxidative addition) tend to have strongly exergonic reductive elimination steps and *vice versa*. These cases are again exemplified by Pd(CO)_2_ (Fig. 2a) with destabilized intermediates and Pd(NH_3_)_2_ (Fig. 2b) with over stabilized intermediates. In contrast to those cases, the thermodynamics of Pd(PMe_3_)_2_ (Fig. 2c) more closely follow the “ideal” line, as expected. Note that the energetics of the oxidative addition and reductive elimination steps more closely align (–24.0 and –27.8 kcal mol^–1^, respectively), which should assist in driving the catalytic cycle forward in a consistent manner.

It is important to remember that this picture only considers the thermodynamics of the catalytic cycle and ignores kinetic aspects. Of course, it is well understood that the difference between a “good” and “bad” catalyst often depends upon the barrier heights associated with movements between intermediates. This is particularly true for establishing reaction enantioselectivity, where the final products depend upon the detailed kinetics of each system. For the purposes of initial characterization of catalysts it is assumed that the system is under thermodynamic control. Within the context of volcano plots, the first priority is validating system thermodynamics, which determine the plausibility of a reaction proceeding for a given catalyst. Since comparable scaling relations between free energies and barrier heights have been identified, similar plots that explicitly incorporate activation barriers could be envisioned. Indeed, Bell–Evans–Polanyi scaling relations, which relate thermodynamics with kinetics, have been used in heterogeneous catalysis^45,46^ and should also be appropriate for homogeneous systems. Alternatively, the kinetics of a handful of systems identified by volcano plots as being the most thermodynamically appealing could be examined in more detail. In this manner, a great deal of time is saved since systems with poor thermodynamics are excluded from the onset.

### Linear free energy scaling relationships

**Fig. 3:**
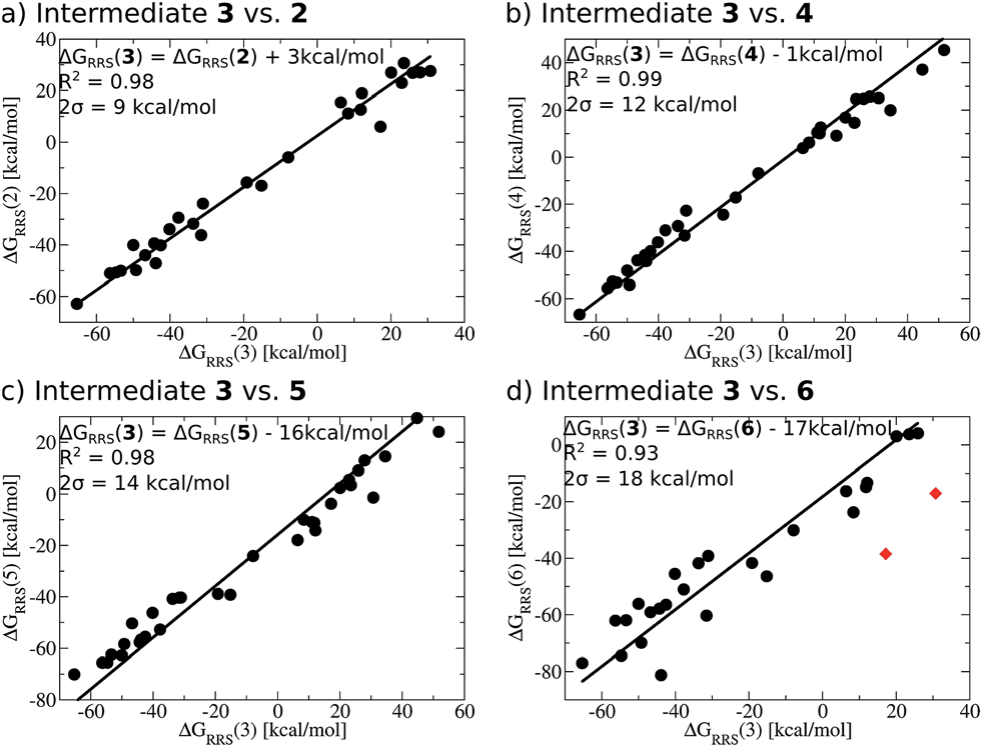
Linear scaling relationships amongst intermediates. Free energies (Δ*G*_RRS_) are relative to the catalytic resting state, as defined by eqn (2)–(6). Equations defining the liner scaling relationships (upper left) are used to create volcano plots (*vide infra*). No comparison between **1** and **3** is possible, since Δ*G*_RRS_(**1**) is, by definition, zero. Comparisons with **3** are equal to unity. Red points in (d) have been excluded from the data fitting equations based on Grubbs' statistical test for outliers. Note that equations derived from the linear scaling relationships appear to be independent of computational level (see ESI† for details).

When a sufficiently large number of catalysts are screened for a particular reaction, it becomes possible to establish whether linear scaling relationships exist. Assuming a sufficiently good correlation, these relationships permit the description of the stability of an intermediate with respect to the relative stability of a descriptor intermediate. For example, Fig. 3a shows that the Δ*G*_RRS_ of intermediates **3** and **2** correlate extremely strongly with one another (*R* ^2^ = 0.98), thereby allowing Δ*G*_RRS_(**2**) to be cast in terms of Δ*G*_RRS_(**3**)^47^ [Δ*G*_RRS_(**3**) = Δ*G*_RRS_(**2**) + 3 kcal mol^–1^]. Aside from the convenient ability to describe the Δ*G* of one intermediate in terms of another, this mathematical relationship also has direct chemical meaning, where the slope of unity indicates similar bonding patterns and the *y*-intercept of +3 kcal mol^–1^ indicates that, on average, the Δ*G*_RRS_(**3**) lies 3 kcal mol^–1^ higher in energy than Δ*G*_RRS_(**2**). Similar extremely strong correlations are seen between Δ*G*_RRS_(**3**) and Δ*G*_RRS_(**4**) (Fig. 3b, *R* ^2^ = 0.99), Δ*G*_RRS_(**5**) (Fig. 3c, *R* ^2^ = 0.98) and Δ*G*_RRS_(**6**) (Fig. 3d, *R* ^2^ = 0.93). Owing to these strong correlations, it becomes possible to describe the average expected value of each intermediates entirely in terms of Δ*G*_RRS_(**3**). The importance of these relationships cannot be over stressed; as these descriptors will later define the volcano plots (*vide infra*). One minor shortcoming is that these scaling relations describe only electronic effects of the system. This current limitation would make description of catalysts with bulky ligands where steric interactions are used to drive reaction enantioselectivity difficult. Accordingly, corrections for such effects should be considered in the future.2
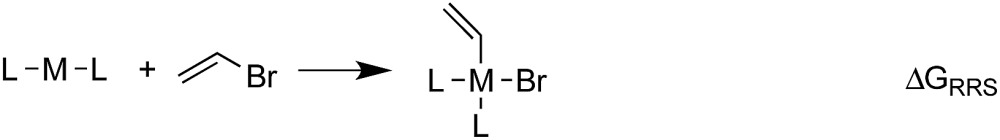

3
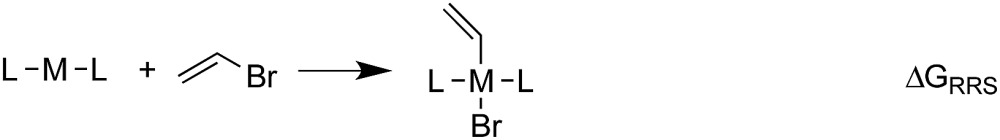

4
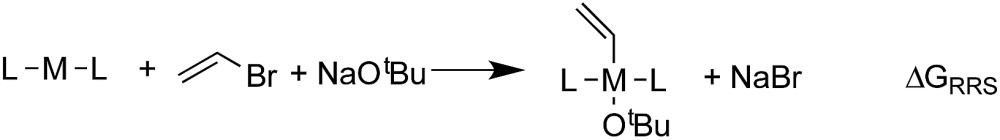

5
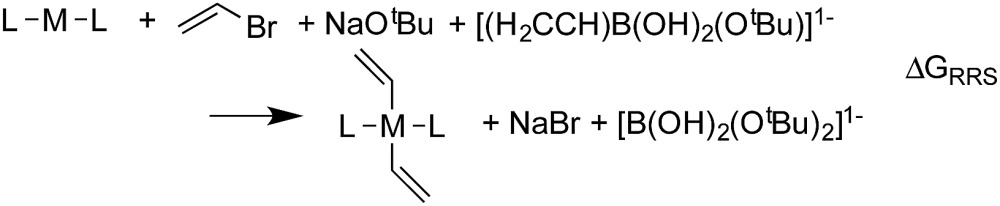

6
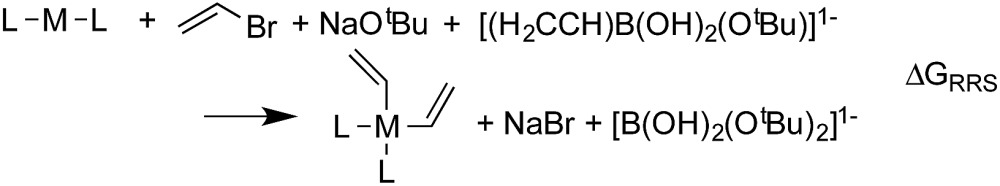



The *y*-intercepts of the linear scaling free energy relationships can further serve to identify any energetically problematic steps between catalytic cycle intermediates. These would be associated with large positive *y*-intercepts, which indicate significant thermodynamic barriers.^48^ Large *y*-intercepts of this type are absent for the Suzuki reaction. The small intercept values given by the relationships between **3** and **2** as well as **3** and **4** indicate that these intermediates, on average, lie energetically near one another. On the other hand, the large negative *y*-intercepts for the linear scaling relationships between **3** and **5** as well as **3** and **6** indicate that these later steps lie significantly lower in energy than intermediate **3**, which should help drive the catalytic cycle toward completion. From the linear scaling relationships derived in Fig. 3 it is expected that this particular reaction should proceed smoothly through the intermediates without any major thermodynamic barrier.

### Construction of volcano plots

**Fig. 4:**
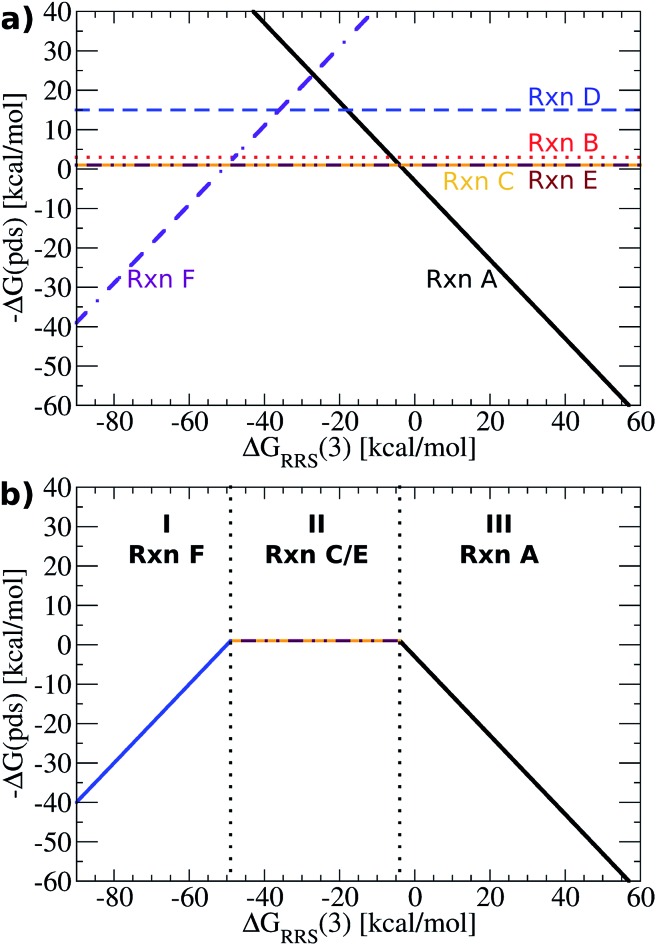
(a) Plot of linear scaling relationships, derived from intermediates ((reactions **B–E**), depicted as straight lines) from the corresponding linear relationships (Fig. 3). Reactions **A** (oxidative addition) and **F** (reductive elimination) appear as sloped lines. (b) Volcano plot derived from (a). Reactions **C** and **E** are the potential determining step for the plateau region. Lines defining the volcano are obtained by taking the lowest –Δ*G*(pds) value amongst all reactions for each Δ*G*_RRS_(**3**) value.

Having established the existence of linear scaling relationships amongst the catalytic cycle intermediates, volcano plots, which aid in the identification of thermodynamically attractive candidates, can be constructed. The basic premise of such plots is to illustrate relationships between the catalytic cycle reaction free energies (Δ*G*_Rxn_, *y*-axis) and the stability of a chosen intermediate species relative to the catalytic resting state (Δ*G*_RRS_, *x*-axis). Lines defining reaction energies are obtained from the previously derived linear scaling relationships (upper left corner of Fig. 3 plots). Because the scaling relation slopes are equivalent for intermediates **2–6** (see Fig. 3), reactions that successively transition between these intermediates (**B–E**) appear as horizontal lines, *i.e.*, the reaction free energy is independent from the choice of catalyst for these steps. Reactions **A** (oxidative addition) and **F** (reductive elimination), however, appear as sloped lines owing to their dependence on Δ*G*_RRS_(**3**) (see Fig. 4a), the descriptor intermediate. Detailed explanations and derivations of the Fig. 4a equations are provided in the ESI.†

While Fig. 4a shows the average reaction free energies (**A–F**) relative to Δ*G*_RRS_(**3**) obtained from the linear scaling relationships, the volcano plots are defined in terms of a “potential determining step” Δ*G*(pds), determined by eqn (7). Pictorially, the reaction line (**A–F**, Fig. 4a) with the most negative (or least positive) –Δ*G*(pds) value for any Δ*G*_RRS_ defines the potential determining step. An examination of Fig. 4a reveals that reaction **F** has the lowest values for Δ*G*_RRS_(**3**) values less than –50 kcal mol^–1^; both reactions **C** (ligand exchange) and **E** (*trans*/*cis* isomerization) have the most negative –Δ*G*(pds) values for Δ*G*_RRS_(**3**) between ∼–50 and ∼–10 kcal mol^–1^; and reaction **A** has the most negative values for Δ*G*_RRS_(**3**) quantities greater than –10 kcal mol^–1^. Taking only the reaction lines with the lowest values for Δ*G*_RRS_(**3**) gives the shape of the volcano plot, Fig. 4b.

7 Δ*G*(pds) = max[Δ*G*_Rxn_(A), Δ*G*_Rxn_(B), Δ*G*_Rxn_(C), Δ*G*_Rxn_(D), Δ*G*_Rxn_(E), Δ*G*_Rxn_(F)]

For characterization purposes, this volcano plot can be subdivided into three sections: the left slope, conventionally referred to as the “strong binding side” (I, Fig. 4b) where intermediates are overly stabilized relative to the “hypothetical ideal catalyst” (*e.g.*, dotted lines, Fig. 2), and reductive elimination (reaction **F**) is potential determining, a “weak binding side” (III, Fig. 4b) with under or destabilized intermediates, making oxidative addition (reaction **A**) potential determining, and the plateau region where the free energies associated with oxidative addition and reductive elimination are roughly balanced (II, Fig. 4b). It is in this final area that catalysts having the most appealing thermodynamic profiles fall. Distinguishing thermodynamically attractive catalysts is then remarkably simple; those with the largest –Δ*G*(pds) values (*e.g.*, higher on or above the volcano) are the most attractive since they have the most exergonic reaction free energy for the potential determining step.

**Fig. 5:**
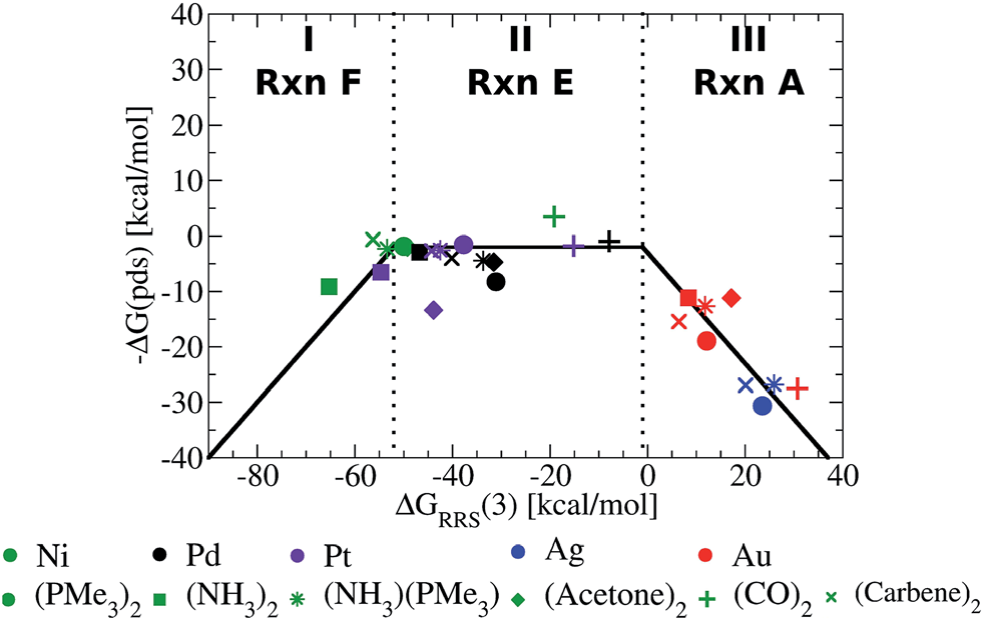
Volcano plot illustrating the thermodynamic suitability of potential catalysts derived from Fig. 4. Reaction **E** is the potential determining step for the plateau region. Lines defining the volcano are obtained by taking the lowest –Δ*G*(pds) value amongst all reactions (**A–F**) for each Δ*G*_RRS_(**3**) value.

Fig. 5 presents the volcano constructed from the linear scaling relationships in Fig. 4b, with points representing the individual catalysts now included. The location of the each catalyst in one of the three defining regions (I–III) is determined solely by its value of Δ*G*_RRS_(**3**), making creation of the final volcano plot quite easy. A closer examination immediately reveals the superior performance of group 10 metal catalysts (Ni, Pd, Pt) relative to those possessing coinage metal centres (Cu, Ag, Au).^49^ The later catalysts appear uniformly on the volcano's “weak binding side”, which aligns with known difficulties involving oxidative addition for gold and silver catalysts.^50^ While it may have been possible to make this prediction in advance based on chemical knowledge and intuition, it is an important point and critical validation of the model that the volcano plot is able to reproduce these experimental observations without requiring any knowledge of behaviour of these catalysts.

While it is simple to characterize differences between catalysts with group 10 and 11 metal centres based on the Fig. 5 volcano, distinguishing amongst the different group 10 metal catalysts is more difficult. It should be noted, however, that several nickel catalysts (depicted in green) are predicted to perform well, which is attractive from the perspective of using earth abundant metals for catalysis.^51^ Ligand influences are also clearly seen. Considering a catalyst on the strong binding side (region I) of the volcano, Ni(NH_3_)_2_, destabilizing the intermediates will shift a Ni catalyst into region II. This can be achieved by replacing ammonia with CO ligands, which succeed in not only destabilizing the intermediates into region II but also increasing the exergonicity of the potential determining step.

**Fig. 6:**
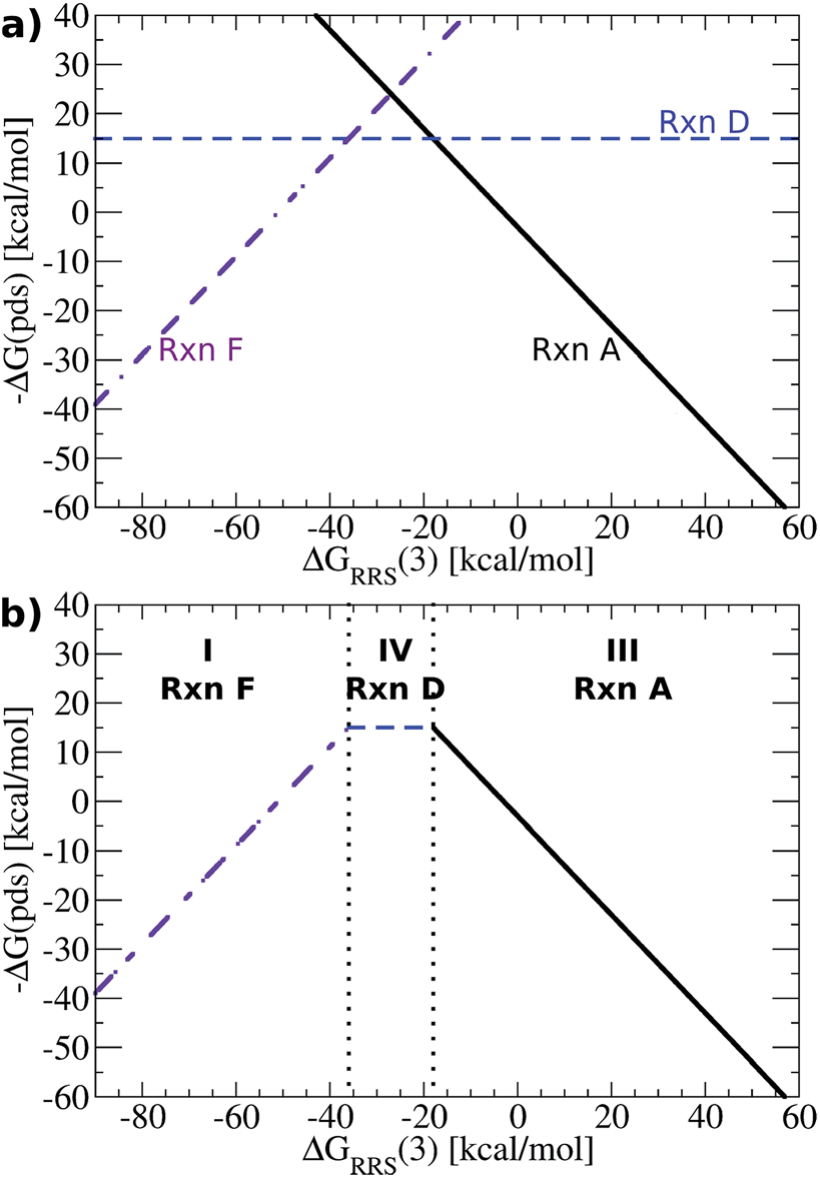
(a) Plot of linear scaling relationships, derived from intermediates where reactions **B**, **C**, and **E** have been removed because they are known to occur rapidly based on experimental observations. Reactions **A** (oxidative addition) and **F** (reductive elimination) appear as sloped lines. (b) Volcano plot derived from (a). Reaction **D** is the potential determining step for the plateau region. Lines defining the volcano are obtained by taking the lowest –Δ*G*(pds) value amongst all reactions for each Δ*G*_RRS_(**3**) value.

**Fig. 7:**
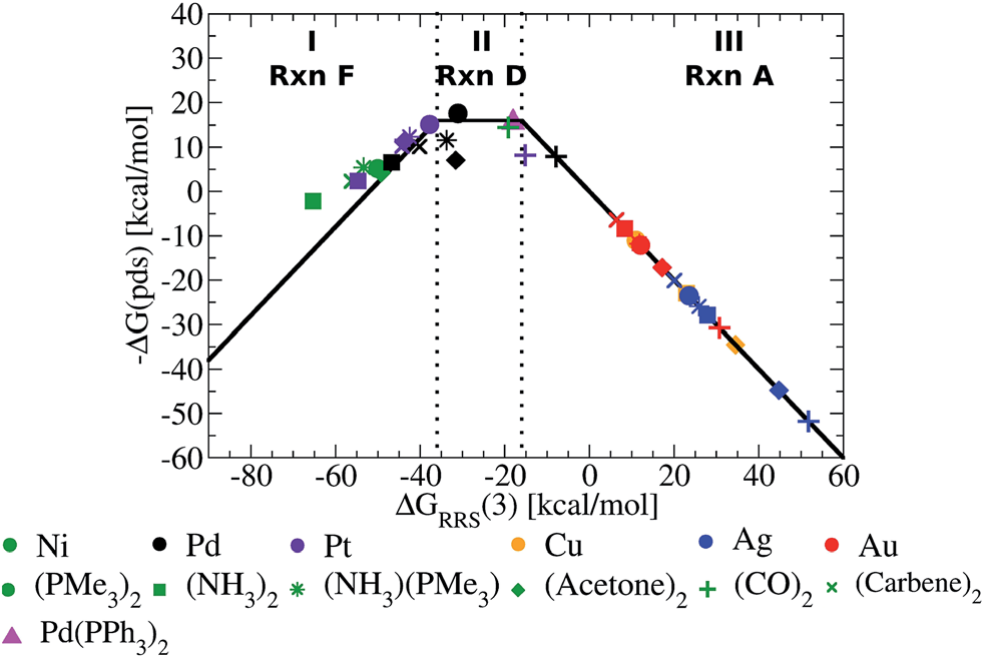
Refined volcano plot illustrating the thermodynamic suitability of potential catalysts where the isomerization and ligand exchange steps are ignored. The most attractive candidates lie near the top of the volcano in region II. Equations used to derive the volcano are presented in the ESI.†

The Fig. 5 volcano plot corresponds to a situation in which only the mechanism of the catalytic cycle is known. Given this same information, volcano plots can be constructed and applied to nearly any reaction from homogeneous catalysis. Of course, chemists are not only interested in developing new reactions, but also frequently search for more efficient, cheaper, or more environmentally friendly catalysts for catalytic processes with rich and well-established chemistries. In these instances a great deal of information regarding, for example, the ease and speed of transformation between different intermediates may have been garnered from experimental studies. Valuable information of this type can also been incorporated into volcano plots and assist in predicting a more refined set of catalytic candidates. For Suzuki coupling the isomerization (reactions **B** and **E**) and ligand exchange (reaction **C**) steps are known to occur relatively rapidly compared to the transmetallation step in the laboratory,^52,53^ meaning that they are highly unlikely to be potential determining for the catalytic cycle. Therefore, it is reasonable to eliminate these as possible potential determining steps and create a new volcano plots in which reactions **B**, **C**, **E** have been removed (Fig. 6, see ESI† for details). This leaves transmetallation (reaction **D**), rather than ligand exchange (reaction **C**) or *cis*/*trans* isomerization (reaction **E**) to define the plateau of the refined volcano (Fig. 6b). The refined volcano plot including points for the individual catalysts in shown in Fig. 7. Here, in comparison to the Fig. 5 volcano plot, the number of catalysts appearing in the thermodynamically “well-balanced” plateau region is greatly reduced. Of these five attractive candidates, three incorporate a palladium centre [Pd(PMe_3_)_2_, Pd(acetone)_2_, Pd(NH_3_) (PMe_3_)], one has a nickel centre, Ni(CO)_2_, and the final is Pd(PPh_3_)_2_, which will be discussed shortly. Gratifyingly, the truncated version of Suzuki's original catalyst is identified as the most attractive thermodynamic candidate, represented by its placement above the plateau. Likewise, Pd(carbene)_2_, while not located in region II of Fig. 7, does lie relatively high on the volcano. The location of this particular species in region I does, however, indicate that reductive elimination may sometimes be problematic for this species. Overall, the high ranking of the phosphine and carbene catalysts further validates the conceptual use of volcano plots for identifying thermodynamically attractive homogeneous catalysts.

Now that concept of using linear scaling relationships to create volcano plots has been demonstrated for homogeneous catalysis, it is important to explain how previously constructed volcano plots could easily be used to determine the viability of new catalysts, particularly for new, less studied reactions. The thermodynamics of a potential catalyst can be assessed by computing the free energies of only four intermediates, which determine the binding (Δ*G*_RRS_) and reaction energies (Δ*G*_RxnA_, *etc.*) necessary to place a catalysts onto the volcano plot. Using Suzuki coupling as an example, it would first be necessary to compute the energies of **1** and **3** (plus vinylbromide) that provides the value of Δ*G*_RRS_(**3**). This value determines which of the potential determining steps [–Δ*G*(pds) for regions I, II, or III, Fig. 7], governs this catalyst. For the sake of argument, let us assume that the Δ*G*_RRS_(**3**) value lies within region II (Fig. 7). It would then be necessary to determine the energies of **4** and **5** (plus the requisite boron compounds) to calculate the –Δ*G*(pds) corresponding to reaction **D**. Δ*G*_RRS_(**3**) values falling in other regions (*e.g.*, I or III) require the determination of the energies of other intermediates. The new catalyst can then be placed onto a previously determined volcano using the Δ*G*_RRS_ and –Δ*G*(pds) as a set of Cartesian coordinates. In this way, the screening of new catalysts represents a significant computational speed up, as it is not necessary to compute the entire catalytic cycle. Moreover, the initial volcano plot can be established using a set of simpler ligands for which the complete catalytic cycle can be computed more rapidly. Catalysts bearing, for example, larger and more exotic ligands, can then be assessed *via* the computationally reduced procedure described directly above.

To illustrate this point, we computed the four necessary intermediates [**1**, **2**, **4**, and **5**, as the Δ*G*_RRS_(**3**) places this catalyst in region II of Fig. 7] necessary to place Pd(PPh_3_)_2_ onto the volcano plot (Fig. 7, pink triangle). As expected, Pd(PPh_3_)_2_ lies high on the volcano, consistent with its known efficacy for Suzuki coupling. We emphasize to determine this there was no need to compute the entire catalytic cycle. This example illustrates the way in which catalytic screening using existing volcano plots constructed based on simpler ligands can proceed. In this manner, it is envisioned that volcano plots based on linear scaling relationships could become a valuable tool for *in silico* catalytic screening.

## Conclusion

We assessed the ability of linear scaling relationships and volcano plots to reproduce known catalytic trends and artefacts for the well-studied Suzuki reaction. This proof-of-principle example shows that these commonly used tools borrowed from the heterogeneous catalysis community succeed in reproducing known trends and observations for homogeneous catalysis. While the aim of this study was to validate and show functionality of the model, in the future studies employing this same methodology have the potential to be extremely helpful for identifying attractive homogeneous catalysts. Constructing volcano plots based on computed data can serve as an important precursor step to the synthesis of new catalysts for myriad chemical reactions through *a priori* computational screening and identification of thermodynamically attractive candidates.
