# Supplementary material for: Linear scaling relationships and volcano plots in homogeneous catalysis – revisiting the Suzuki reaction
Source: Chem Sci. 2015 Sep 2;6(12):6754–61. doi: 10.1039/c5sc02910d (PMC5508671; doi:10.1039/c5sc02910d)
Supplement: Supplementary file 1 [file SC-006-C5SC02910D-s001.pdf]

Electronic Supplementary Material (ESI) for Chemical Science.
This journal is © The Royal Society of Chemistry 2015

Supplementary Information

Linear Scaling Relationships and Volcano Plots in Homogeneous Catalysis – Revisiting the Suzuki Reaction

Michael Busch, Matthew D. Wodrich, and Clémence Corminboeuf

Linear Scaling Relations and Volcano Plots

Volcano plots summarize the relative activity of a candidate catalyst with respect to a descriptor. It is important to emphasis that in principle any quantity related to the activity of the candidate catalysts may be used. This includes but is not restricted to the energetics of reaction steps or an intermediate's free energy relative to the resting state. In the present case the stability of intermediate **3** ΔG_{RRS}(**3**) has been chosen as it comprises the central precursor for the transmetallation step. Since a purely thermodynamic perspective on the Suzuki coupling is employed the thermodynamically least favorable step is used to describe the relative activity of the candidate catalysts. This potential determining step ΔG(pds) is given by:

$$\Delta G(pds) = \max[\Delta G_{Rxn}(\mathbf{A}), \Delta G_{Rxn}(\mathbf{B}), \Delta G_{Rxn}(\mathbf{C}), \Delta G_R xn(\mathbf{D}), \Delta G_{Rxn}(\mathbf{E}), \Delta G_{Rxn}(\mathbf{F})] \quad \text{(Equation S1)}$$

where Rxns **A**-**F** are defined in Figure 1 of the manuscript. A central part of any volcano plot is the comparison of the computed reaction energies and their relative position in the volcano as indicated by the different slopes (see lines in figures 4 and 5 in the paper). These slopes summarize the theoretical energetics of the potential determining step and are obtained purely from linear scaling relationships.

Linear scaling relations are derived assuming the reactants as reference states

$$G(\mathbf{1}) = G(CH_2CHBr) = G(NaOtBu) = G([(CH_2CH)B(OH)_2OtBu]^-) = 0kcal/mol \quad \text{(Equation S2)}$$

while the energy of product 1,3-Butadiene formed corresponds to the energy of the total reaction.

$$\Delta G(1,3 - Butadiene) = -68kcal/mol \quad \text{(Equation S3)}$$

The energetics for the conversion of NaOtBu into NaBr during ligand exchange (Reaction **C**) and of $[(CH_2CH)B(OH)_2OtBu]^-$ into $[B(OH)_2(OtBu)_2]^-$ during the transmetallation step (Reaction **D**) are taken from DFT calculations. Based on these assumptions the following sets of scaling relations between the free energies of key intermediates **r**elative to the **r**esting **s**tate (ΔG_{RRS}) were obtained for the Suzuki coupling (see Figure 3 in the paper):

$$\Delta G_{RRS}(\mathbf{2}) = \Delta G_{RRS}(\mathbf{3}) + 3kcal/mol \quad \text{(Equation S4)}$$

$$\Delta G_{RRS}(\mathbf{3}) = \Delta G_{RRS}(\mathbf{3}) \quad \text{(Equation S5)}$$

$$\Delta G_{RRS}(\mathbf{4}) = \Delta G_{RRS}(\mathbf{3}) - 1kcal/mol \quad \text{(Equation S6)}$$

$$\Delta G_{RRS}(\mathbf{5}) = \Delta G_{RRS}(\mathbf{3}) - 16kcal/mol \quad \text{(Equation S7)}$$

$$\Delta G_{RRS}(\mathbf{6}) = \Delta G_{RRS}(\mathbf{3}) - 17kcal/mol \quad \text{(Equation S8)}$$

Having established scaling relations between the key intermediates the theoretical reaction energies can be calculated as follows:

1. **Reaction A: Oxidative Addition**

The first reaction step of the Suzuki coupling comprises the oxidative addition of CH_2CHBr to the catalyst:

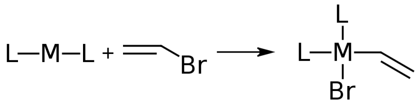

(Equation S9)

According to reaction S9 the theoretical reaction free energy ΔG_{theo} for this reaction is given by:

$$-\Delta G_{theo}(\mathbf{A}) = -[\Delta G_{RRS}(\mathbf{2}) - (G(\mathbf{1}) + G(EtBr))] \quad \text{(Equation S10)}$$

Taking ΔG_{RRS}(**2**) from equation S4 and G(**1**) and G(CH_2CHBr) from equation S2 one obtains:

$$-\Delta G_{theo}(\mathbf{A}) = -\Delta G_{RRS}(\mathbf{3}) - 3kcal/mol \quad \text{(Equation S11)}$$

2. **Reaction B: cis-trans Isomerization**

The isomerization from cis to trans corresponds to:

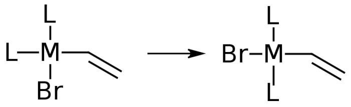

(Equation S12)

Accordingly the reaction free energy is given by:

$$-\Delta G_{theo}(\mathbf{B}) = -[\Delta G_{RRS}(\mathbf{3}) - \Delta G_{RRS}(\mathbf{2})] \quad \text{(Equation S13)}$$

With the scaling relations for intermediate **2** (equation S4) and **3** (equation S5) one obtains:

$$-\Delta G_{theo}(\mathbf{B}) = 3kcal/mol \quad \text{(Equation S14)}$$

3. **Reaction C: Ligand Exchange**

Exchange of a Br ligand for a OtBu group is modeled with respect to “molecular” NaBr and NaOtBu.

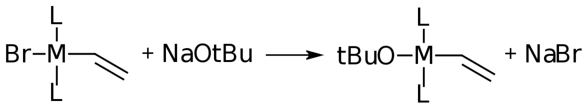

(Equation S15)

The reaction free energy is thus given by the scaling relations of the involved intermediates as these already include the conversion of NaOtBu to NaBr.

$$-\Delta G_{theo}(\mathbf{C}) = -[\Delta G_{RRS}(\mathbf{4}) - \Delta G_{RRS}(\mathbf{3})] \quad \text{(Equation S16)}$$

Inserting the relevant scaling relations from equations S5 and S6 one obtains:

$$-\Delta G_{theo}(\mathbf{C}) = 1kcal/mol \quad \text{(Equation S17)}$$

4. **Reaction D: Transmetallation**

Ligand exchange is followed by transmetallation:

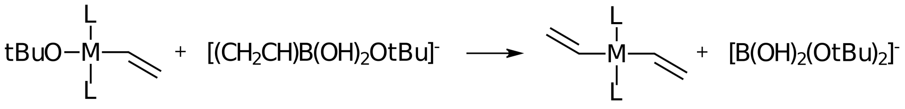

(Equation S18)

The reaction free energies for this central reaction step are given by:

$$-\Delta G_{theo}(\mathbf{D}) = -[\Delta G_{RRS}(\mathbf{5}) - \Delta G_{RRS}(\mathbf{4})] \qquad \text{(Equation S19)}$$

Taking the linear scaling relations for the intermediates **4** and **5** from equations S6 and S7 one obtains:

$$-\Delta G_{theo}(\mathbf{D}) = 15 kcal/mol \qquad \text{(Equation S20)}$$

5. Reaction E: trans-cis Isomerization

The second isomerization step corresponds to:

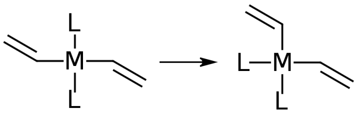

(Equation S21)

Accordingly the theoretical reaction free energy is given by:

$$-\Delta G_{theo}(\mathbf{E}) = -[\Delta G_{RRS}(\mathbf{6}) - \Delta G_{RRS}(\mathbf{5})] \qquad \text{(Equation S22)}$$

With the relevant scaling relations of the intermediates **5** (equation S7) and **6** (equation S8) one obtains:

$$-\Delta G_{theo}(\mathbf{E}) = 1 kcal/mol \qquad \text{(Equation S23)}$$

6. Reaction F: Reductive Elimination

The final reaction step corresponds to the reductive elimination of the 1,3-Butadiene formed as product.

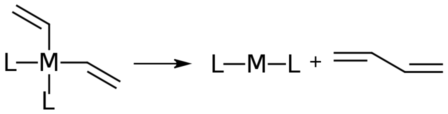

(Equation S24)

Accordingly the theoretical reaction free energy of the final step is given by:

$$-\Delta G_{theo}(\mathbf{F}) = -[(\Delta G_{RRS}(\mathbf{1}) + \Delta G(1,3-Butadiene)) - \Delta G_{RRS}(\mathbf{6})] \qquad \text{(Equation S25)}$$

Taking the scaling relation for intermediate 6 from equation 8, the relative energies of the reactant 1 from equation 2 and 1,3-Butadiene from equation 3 the theoretical reaction free energy is given by:

$$-\Delta G_{theo}(\mathbf{F}) = \Delta G_{RRS}(\mathbf{3}) + 51 kcal/mol \qquad \text{(Equation S26)}$$

In figure S1 a summary of the energetics of the different reaction steps employing the equations S11, S14, S17, S20, S23 and S26 is shown. In this purely thermodynamic analysis the activity of a catalyst is characterized by the thermodynamically least favorable step (see equation S1). It is important to note that explicitly computed reaction energies enter the theoretical reaction free energies used in the volcano shown below only indirectly through the linear scaling relationships.

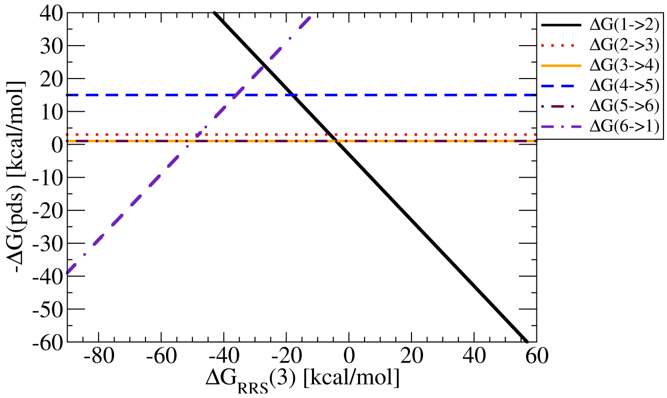

Figure S1: A theoretical volcano derived from linear scaling relations is depicted. The thermodynamically least favorable step corresponds to ΔG(pds) shown in figures 4 and 5 of the paper.

In the case of strongly bound intermediates (i.e. ΔG_{RRS}(**3**) << 0) the reductive elimination step is potential determining (Reaction **F**) while it is the oxidative addition for a weakly bound intermediate **3** (ΔG_{RRS}(**3**) > 0 or ΔG_{RRS}(**3**) ≈ 0). For intermediate situations trans-cis isomerization (Reaction **E**) or when neglecting isomerization and ligand exchange steps the transmetallation (Reaction **D**) are potential determining.

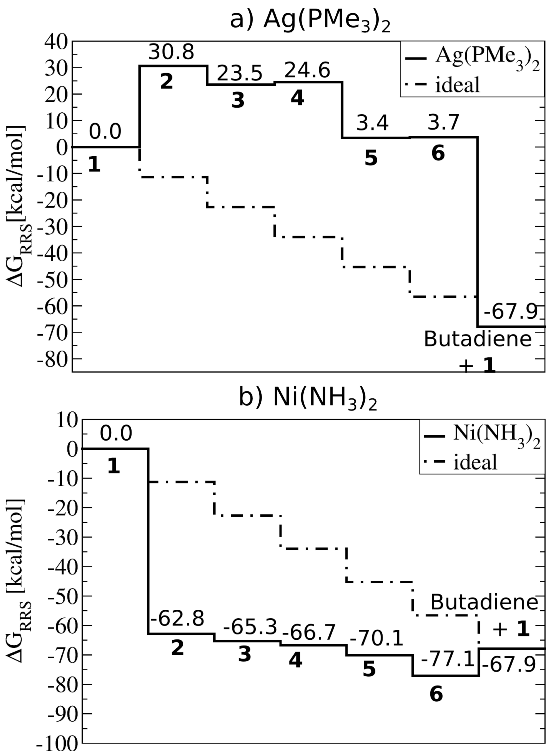

Figure S2: Free energy plots for "extreme" examples that have endergonic oxidative addition (a) or reductive elimination (b) steps.

Scaling Relationships (M06/def2-SVP)

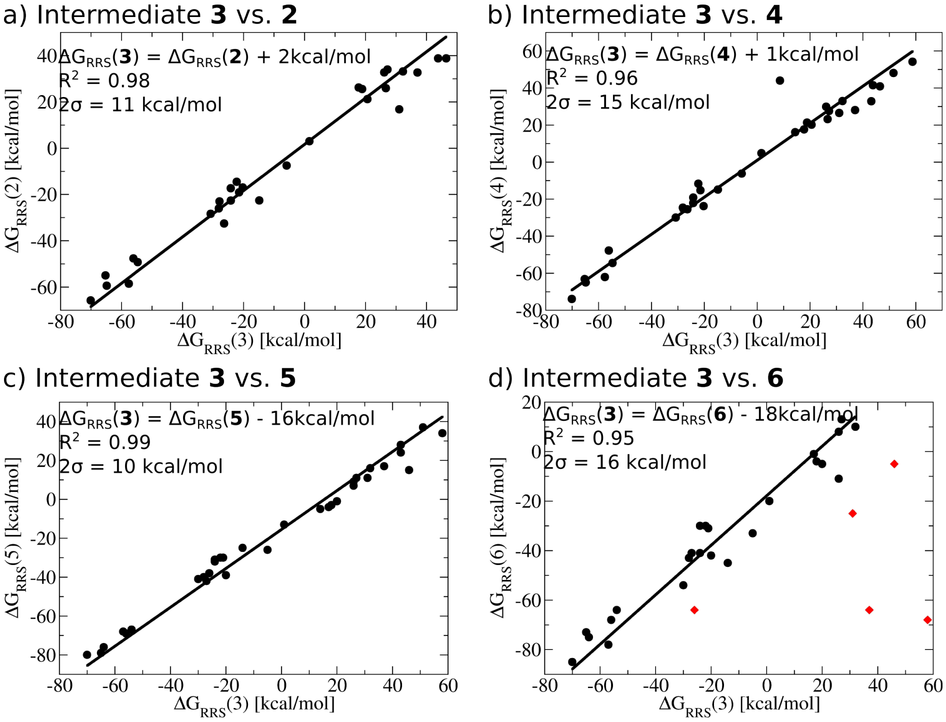

Figure S3: The scaling relationships obtained from Gaussian09 calculations (M06/def2-SVP) are depicted. Excellent agreement with the results shown in the paper is found.

Functional Dependence of Results

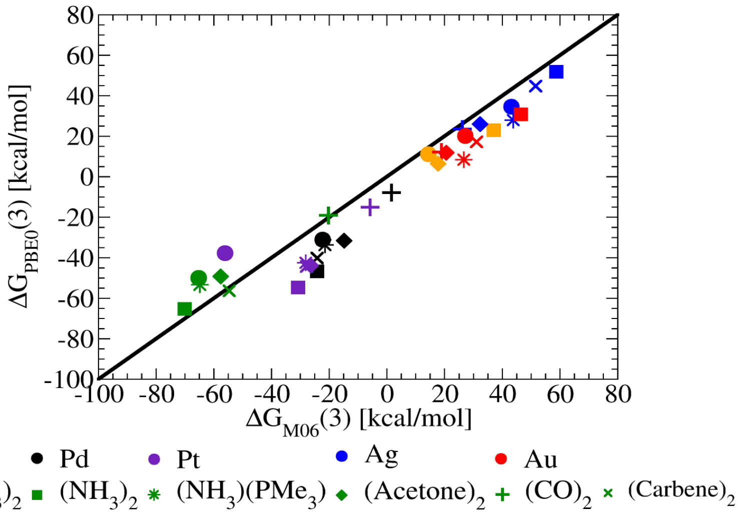

*Figure S4: A comparison between the binding energies of intermediate **3** obtained with PBE0-dDsC/TZ2P//M06/def2-SVP and M06/def2-SVP is depicted.*

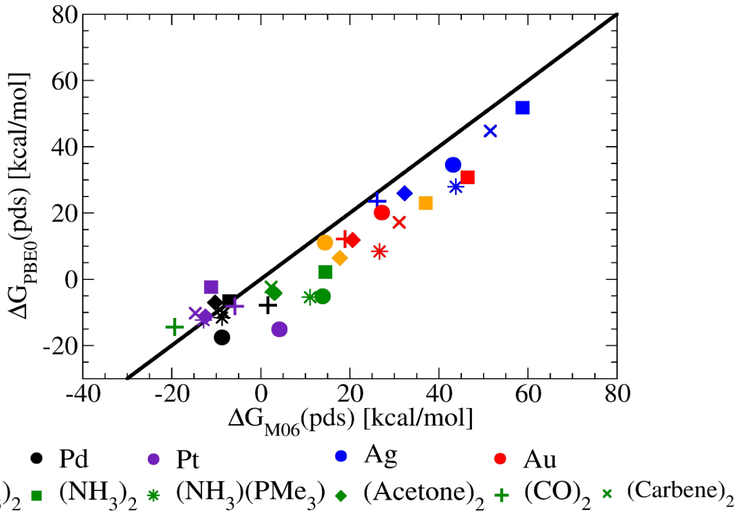

Figure S5: A comparison between the free energy of the potential determining step (pds) obtained withPBE0-dDsC/TZ2P//M06/def2-SVP and M06/def2-SVP is depicted.

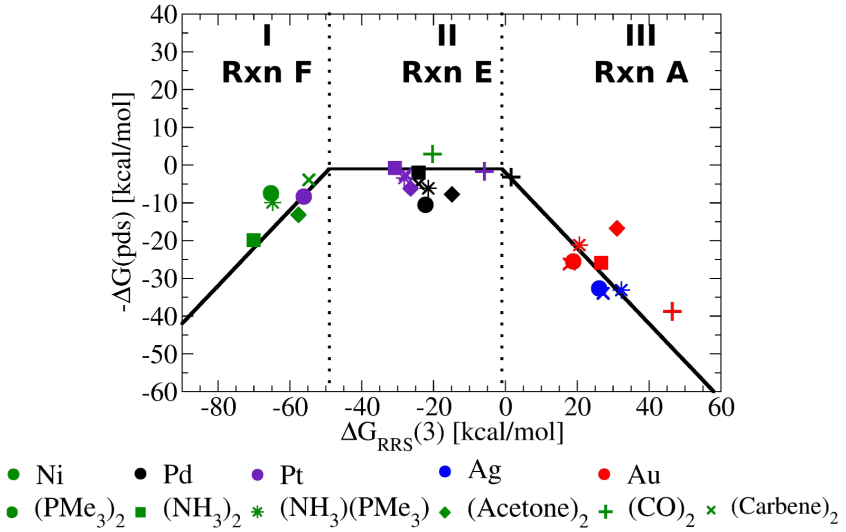

Figure S6: The volcano plot obtained from Gaussian09 calculations (M06/def2-SVP) assuming the full Suzuki mechanism is depicted.

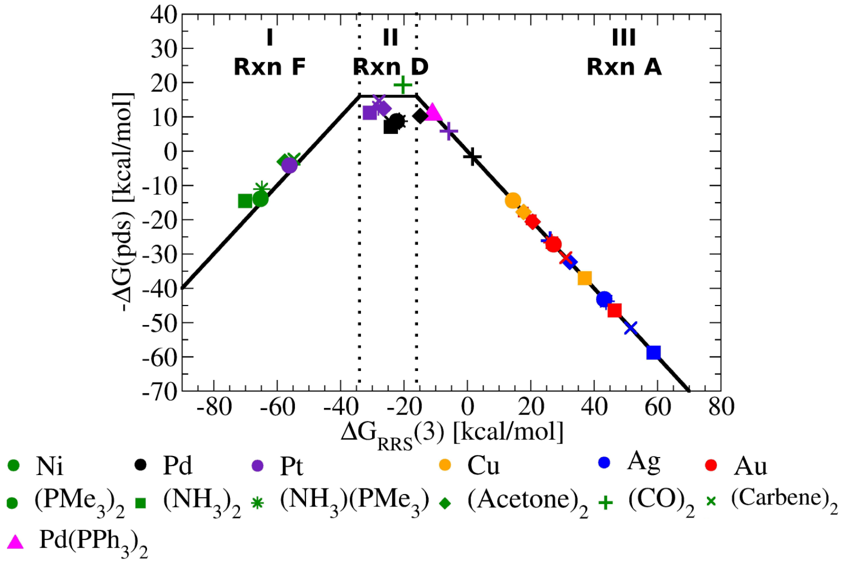

Figure S7: The volcano plot obtained from Gaussian09 calculations (M06/def2-SVP) assuming the reduced Suzuki mechanism is depicted.

Summary of Computed Free Energies Relative to Resting State ΔG_{RRS}

Catalyst	$\Delta G_{RRS}(2)$	$\Delta G_{RRS}(3)$	$\Delta G_{RRS}(4)$	$\Delta G_{RRS}(5)$	$\Delta G_{RRS}(6)$
$Ni(CO)_2$	-15.70	-19.15	-24.46	-38.90	-41.73
$Ni(NH_3)_2$	-62.85	-65.30	-66.73	-70.13	-77.07
$Ni(acetone)_2$	-49.88	-49.24	-54.23	-58.42	-69.92
$Ni(carbene)_2$	-51.06	-56.29	-55.65	-65.60	-62.13
$Ni(PMe_3)_2$	-40.08	-50.00	-48.15	-62.77	-56.18
$Ni(PMe_3)(NH_3)$	-50.10	-53.38	-53.24	-62.51	-61.97
$Pd(CO)_2$	-5.94	-7.86	-6.88	-24.15	-30.10
$Pd(NH_3)_2$	-44.04	-46.74	-43.83	-50.41	-59.16
$Pd(acetone)_2$	-36.26	-31.55	-33.34	-40.39	-60.35
$Pd(carbene)_2$	-33.94	-40.15	-36.16	-46.29	-45.55
$Pd(PMe_3)_2$	-23.98	-31.06	-22.78	-40.34	-39.18
$Pd(PMe_3)(NH_3)$	-31.79	-33.70	-29.26	-40.83	-41.79
$Pt(CO)_2$	-16.98	-15.16	-17.15	-39.25	-46.43
$Pt(NH_3)_2$	-50.76	-54.69	-52.78	-65.56	-74.46
$Pt(acetone)_2$	-47.15	-43.87	-44.24	-56.70	-81.28
$Pt(carbene)_2$	-39.44	-44.29	-41.62	-57.71	-57.86
$Pt(PMe_3)_2$	-29.44	-37.72	-31.09	-52.80	-51.04
$Pt(PMe_3)(NH_3)$	-40.22	-42.50	-39.94	-55.59	-56.49
$Cu(CO)_2$	--	--	30.54	--	--
$Cu(NH_3)_2$	22.92	22.97	14.58	5.54	--
$Cu(acetone)_2$	--	34.54	19.91	14.50	--
$Cu(carbene)_2$	--	--	3.83	-11.30	-8.41
$Cu(PMe_3)_2$	--	11.05	10.48	-10.91	--
$Cu(PMe_3)(NH_3)$	--	--	11.16	-2.43	--
$Ag(CO)_2$	--	51.78	45.28	24.06	--
$Ag(NH_3)_2$	27.02	27.94	25.61	12.93	--
$Ag(acetone)_2$	--	44.79	36.99	29.43	--
$Ag(carbene)_2$	26.95	20.09	16.80	2.38	2.99
$Ag(PMe_3)_2$	30.64	23.55	24.56	3.42	3.71
$Ag(PMe_3)(NH_3)$	26.82	25.93	24.68	9.11	4.08
$Au(CO)_2$	27.53	30.75	25.01	-1.40	-17.06
$Au(NH_3)_2$	11.16	8.40	6.17	-10.05	-23.74
$Au(acetone)_2$	6.02	17.19	9.09	-3.79	-38.49
$Au(carbene)_2$	15.43	6.41	3.82	-17.94	-16.30
$Au(PMe_3)_2$	18.93	12.14	12.58	-14.20	-13.32
$Au(PMe_3)(NH_3)$	12.65	11.81	10.11	-11.23	-14.81

*Table S1: Computed free energies relative to the resting state (**1**) for each intermediate. Values in kcal/mol. Computations at the PBE0-dDsC/TZ2P//M06/def2-SVP level including unscaled free energy correction (M06/def2-SVP) and solvation correction (in THF) from COSMO-RS.*

Computed Free Energies for Relevant Reactions

Catalyst	ΔG_{Rxn}(A)	ΔG_{Rxn}(B)	ΔG_{Rxn}(C)	ΔG_{Rxn}(D)	ΔG_{Rxn}(E)	ΔG_{Rxn}(F)
$Ni(CO)_2$	-15.70	-3.44	-5.32	-14.44	-2.82	-26.20
$Ni(NH_3)_2$	-62.85	-2.45	-1.43	-3.40	-6.95	9.15
$Ni(acetone)_2$	-49.88	0.64	-4.99	-4.19	-11.51	2.00
$Ni(carbene)_2$	-51.06	-5.23	0.64	-9.95	3.48	-5.80
$Ni(PMe_3)_2$	-40.08	-9.92	1.86	-14.62	6.59	-11.75
$Ni(PMe_3)(NH_3)$	-50.10	-3.29	0.14	-9.27	0.54	-5.96
$Pd(CO)_2$	-5.94	-1.92	0.97	-17.27	-5.94	-37.82
$Pd(NH_3)_2$	-44.04	-2.69	2.91	-6.58	-8.75	-8.76
$Pd(acetone)_2$	-36.26	4.71	-1.79	-7.05	-19.96	-7.57
$Pd(carbene)_2$	-33.94	-6.21	3.99	-10.13	0.73	-22.37
$Pd(PMe_3)_2$	-23.98	-7.08	8.27	-17.55	1.16	-28.74
$Pd(PMe_3)(NH_3)$	-31.79	-1.91	4.43	-11.57	-0.96	-26.13
$Pt(CO)_2$	-16.98	1.82	-1.99	-22.10	-7.18	-21.49
$Pt(NH_3)_2$	-50.76	-3.93	1.91	-12.78	-8.89	6.53
$Pt(acetone)_2$	-47.15	3.29	-0.38	-12.46	-24.58	13.36
$Pt(carbene)_2$	-39.44	-4.85	2.67	-16.08	-0.16	-10.06
$Pt(PMe_3)_2$	-29.44	-8.28	6.63	-21.71	1.76	-16.88
$Pt(PMe_3)(NH_3)$	-40.22	-2.29	2.56	-15.65	-0.90	-11.43
$Ag(carbene)_2$	26.95	-6.86	-3.29	-14.43	0.61	-70.91
$Ag(PMe_3)_2$	30.64	-7.09	1.01	-21.13	0.29	-71.63
$Ag(PMe_3)(NH_3)$	26.82	-0.89	-1.25	-15.57	-5.03	-72.01
$Au(CO)_2$	27.53	3.22	-5.74	-26.41	-15.66	-50.87
$Au(NH_3)_2$	11.16	-2.76	-2.23	-16.22	-13.70	-44.18
$Au(acetone)_2$	6.02	11.18	-8.11	-12.88	-34.70	-29.43
$Au(carbene)_2$	15.43	-9.02	-2.59	-21.76	1.64	-51.62
$Au(PMe_3)_2$	18.93	-6.79	0.43	-26.78	0.88	-54.60
$Au(PMe_3)(NH_3)$	12.65	-0.84	-1.71	-21.33	-3.59	-53.11

Table S2: Computed free energies for relevant reactions (defined by Figure 1 in the main text). Values in kcal/mol. Computations at the PBE0-dDsC/TZ2P//M06/def2-SVP level including unscaled free energy correction (M06/def2-SVP) and solvation correction (in THF) from COSMO-RS.

Summary of Total Energies, Free Energy Corrections and Solvation Corrections

Species	M06/def2-SVP Electronic Energy	M06/def2-SVP Free Energy Correction	PBE0-dDsC/TZ2P Electronic Energy	COSMO-RS Solvation Energy (PBE0-dDsC/TZ2P)
Ethenylbromide	-2651.612892	0.014763	-1.346128	-0.005716
$B(OH)_2(O^tBu)(Et)^{1-}$	-487.136196	0.160606	-6.105903	-0.079750
$B(OH)_2(O^tBu)_2^{1-}$	-642.052870	0.240450	-8.306996	-0.078286
NaBr	-2736.064074	-0.023180	-0.203839	-0.029715
NaOtBu	-394.993061	0.087693	-3.454385	-0.024398
Butadiene	-155.742180	0.058111	-2.512689	-0.005314
$Ni(CO)_2$ - 1	-1734.454528	-0.014180	-1.682304	-0.005638
$Ni(CO)_2$ - 2	-4386.114226	0.020293	-3.071326	-0.013195
$Ni(CO)_2$ - 3	-4386.118155	0.019032	-3.076650	-0.012096
$Ni(CO)_2$ - 4	-2045.058650	0.135765	-6.336073	-0.012233
$Ni(CO)_2$ - 5	-1890.170323	0.059118	-4.166256	-0.008631
$Ni(CO)_2$ - 6	-1890.174001	0.058168	-4.169025	-0.009411
$Ni(NH_3)_2$ - 1	-1621.011161	0.046461	-1.976105	-0.011083
$Ni(NH_3)_2$ - 2	-4272.749924	0.083434	-3.412103	-0.049297
$Ni(NH_3)_2$ - 3	-4272.757069	0.083666	-3.429620	-0.035922
$Ni(NH_3)_2$ - 4	-1931.699163	0.201593	-6.691239	-0.028861
$Ni(NH_3)_2$ - 5	-1776.793087	0.122336	-4.497959	-0.028510
$Ni(NH_3)_2$ - 6	-1776.800194	0.120862	-4.498035	-0.038031
$Ni(acetone)_2$ - 1	-1893.817877	0.127735	-5.209933	-0.013317
$Ni(acetone)_2$ - 2	-4545.543735	0.162159	-6.643714	-0.030526
$Ni(acetone)_2$ - 3	-4545.544515	0.164433	-6.648617	-0.026880
$Ni(acetone)_2$ - 4	-2204.489268	0.283933	-9.914856	-0.022451
$Ni(acetone)_2$ - 5	-2049.584014	0.204886	-7.727169	-0.017976
$Ni(acetone)_2$ - 6	-2049.597842	0.202620	-7.735116	-0.026102
$Ni(carbene)_2$ - 1	-2276.289379	0.355634	-11.907828	-0.022101
$Ni(carbene)_2$ - 2	-4928.006343	0.395963	-13.350308	-0.038404
$Ni(carbene)_2$ - 3	-4928.013874	0.394844	-13.364553	-0.031376
$Ni(carbene)_2$ - 4	-2586.950403	0.513483	-16.621680	-0.026222
$Ni(carbene)_2$ - 5	-2432.055245	0.433733	-14.437597	-0.026625
$Ni(carbene)_2$ - 6	-2432.051552	0.436233	-14.429742	-0.031442
$Ni(PMe_3)_2$ - 1	-2429.642234	0.177750	-6.060611	-0.012748
$Ni(PMe_3)_2$ - 2	-5081.367384	0.219751	-7.489614	-0.026703
$Ni(PMe_3)_2$ - 3	-5081.382575	0.218550	-7.516622	-0.014306
$Ni(PMe_3)_2$ - 4	-2740.315174	0.336338	-10.761783	-0.018331
$Ni(PMe_3)_2$ - 5	-2585.427378	0.259231	-8.598206	-0.008312
$Ni(PMe_3)_2$ - 6	-2585.418123	0.260204	-8.576859	-0.020128
$Ni(PMe_3)(NH_3)$ - 1	-2025.331507	0.112661	-4.020903	-0.016788
$Ni(PMe_3)(NH_3)$ - 2	-4677.063069	0.151425	-5.457140	-0.036229
$Ni(PMe_3)(NH_3)$ - 3	-4677.070252	0.149947	-5.471850	-0.025276
$Ni(PMe_3)(NH_3)$ - 4	-2336.008083	0.269363	-8.728118	-0.022559
$Ni(PMe_3)(NH_3)$ - 5	-2181.110766	0.190187	-6.547382	-0.019105
$Ni(PMe_3)(NH_3)$ - 6	-2181.108549	0.189875	-6.535090	-0.030218
$Pd(CO)_2$ - 1	-354.314077	-0.013015	-1.547454	-0.002238
$Pd(CO)_2$ - 2	-3005.938268	0.017844	-2.912931	-0.014162
$Pd(CO)_2$ - 3	-3005.937534	0.014880	-2.913369	-0.013818
$Pd(CO)_2$ - 4	-664.870337	0.134694	-6.166509	-0.013301
$Pd(CO)_2$ - 5	-509.983907	0.056270	-4.000185	-0.008936
$Pd(CO)_2$ - 6	-509.994420	0.055709	-4.008026	-0.010005
$Pd(NH_3)_2$ - 1	-240.909806	0.047135	-1.845281	-0.017639
$Pd(NH_3)_2$ - 2	-2892.576577	0.079672	-3.256389	-0.046339
$Pd(NH_3)_2$ - 3	-2892.580038	0.080740	-3.266471	-0.041615

$Pd(NH_3)_2$ - 4	-551.511977	0.197722	-6.520469	-0.034325
$Pd(NH_3)_2$ - 5	-396.612117	0.120288	-4.334322	-0.033736
$Pd(NH_3)_2$ - 6	-396.628001	0.119827	-4.345132	-0.036409
$Pd(acetone)_2$ - 1	-513.701348	0.125475	-5.069264	-0.016779
$Pd(acetone)_2$ - 2	-3165.369847	0.159789	-6.486403	-0.028823
$Pd(acetone)_2$ - 3	-3165.359416	0.161746	-6.480942	-0.028727
$Pd(acetone)_2$ - 4	-824.294502	0.278676	-9.740785	-0.023030
$Pd(acetone)_2$ - 5	-669.394284	0.199019	-7.550863	-0.024743
$Pd(acetone)_2$ - 6	-669.427822	0.199381	-7.581842	-0.025941
$Pd(carbene)_2$ - 1	-896.167650	0.355101	-11.782466	-0.025058
$Pd(carbene)_2$ - 2	-3547.833591	0.395306	-13.196559	-0.042335
$Pd(carbene)_2$ - 3	-3547.840164	0.390954	-13.209403	-0.035035
$Pd(carbene)_2$ - 4	-1206.768853	0.509441	-16.461411	-0.029513
$Pd(carbene)_2$ - 5	-1051.876522	0.432550	-14.281580	-0.028809
$Pd(carbene)_2$ - 6	-1051.874477	0.434400	-14.276551	-0.034519
$Pd(PMe_3)_2$ - 1	-1049.550867	0.179911	-5.951599	-0.014030
$Pd(PMe_3)_2$ - 2	-3701.209337	0.217026	-7.348323	-0.029709
$Pd(PMe_3)_2$ - 3	-3701.220496	0.215964	-7.366198	-0.022057
$Pd(PMe_3)_2$ - 4	-1360.142959	0.337098	-10.609889	-0.020674
$Pd(PMe_3)_2$ - 5	-1205.255857	0.256182	-8.440441	-0.017392
$Pd(PMe_3)_2$ - 6	-1205.254907	0.255723	-8.434217	-0.021316
$Pd(PMe_3)(NH_3)$ - 1	-645.235108	0.111692	-3.905362	-0.017462
$Pd(PMe_3)(NH_3)$ - 2	-3296.900642	0.148640	-5.310979	-0.036528
$Pd(PMe_3)(NH_3)$ - 3	-3296.903533	0.147795	-5.321332	-0.028376
$Pd(PMe_3)(NH_3)$ - 4	-955.830635	0.266654	-8.571233	-0.024627
$Pd(PMe_3)(NH_3)$ - 5	-800.937838	0.186942	-6.393503	-0.021296
$Pd(PMe_3)(NH_3)$ - 6	-800.940594	0.187551	-6.387259	-0.029679
$Pt(CO)_2$ - 1	-345.779389	-0.016621	-1.711297	-0.002351
$Pt(CO)_2$ - 2	-2997.419738	0.018516	-3.100326	-0.012601
$Pt(CO)_2$ - 3	-2997.415398	0.016836	-3.095530	-0.012821
$Pt(CO)_2$ - 4	-656.351672	0.134391	-6.350393	-0.013036
$Pt(CO)_2$ - 5	-501.468766	0.055439	-4.190741	-0.009171
$Pt(CO)_2$ - 6	-501.483418	0.058771	-4.204868	-0.009819
$Pt(NH_3)_2$ - 1	-232.370577	0.045791	-1.995033	-0.025185
$Pt(NH_3)_2$ - 2	-2884.048653	0.080514	-3.421897	-0.051023
$Pt(NH_3)_2$ - 3	-2884.054750	0.082798	-3.433616	-0.047848
$Pt(NH_3)_2$ - 4	-542.989059	0.200225	-6.689434	-0.040767
$Pt(NH_3)_2$ - 5	-388.093880	0.120512	-4.510650	-0.040422
$Pt(NH_3)_2$ - 6	-388.111700	0.120688	-4.523422	-0.042000
$Pt(acetone)_2$ - 1	-505.148345	0.126383	-5.207556	-0.018553
$Pt(acetone)_2$ - 2	-3156.832177	0.160179	-6.642324	-0.029806
$Pt(acetone)_2$ - 3	-3156.823409	0.161293	-6.638470	-0.029536
$Pt(acetone)_2$ - 4	-815.761289	0.282433	-9.899386	-0.024718
$Pt(acetone)_2$ - 5	-660.865188	0.202013	-7.717605	-0.026144
$Pt(acetone)_2$ - 6	-660.905407	0.200580	-7.754420	-0.027074
$Pt(carbene)_2$ - 1	-887.643767	0.355894	-11.979919	-0.006110
$Pt(carbene)_2$ - 2	-3539.318121	0.395441	-13.384365	-0.041148
$Pt(carbene)_2$ - 3	-3539.324106	0.393687	-13.397358	-0.034126
$Pt(carbene)_2$ - 4	-1198.257640	0.513108	-16.651775	-0.029236
$Pt(carbene)_2$ - 5	-1043.368536	0.433419	-14.478251	-0.028916
$Pt(carbene)_2$ - 6	-1043.366658	0.433970	-14.473545	-0.034420
$Pt(PMe_3)_2$ - 1	-1041.018869	0.177111	-6.116685	-0.016269
$Pt(PMe_3)_2$ - 2	-3692.688090	0.219490	-7.530154	-0.029175
$Pt(PMe_3)_2$ - 3	-3692.698610	0.216501	-7.547181	-0.022352
$Pt(PMe_3)_2$ - 4	-1351.623547	0.336630	-10.792644	-0.020815
$Pt(PMe_3)_2$ - 5	-1196.742974	0.257648	-8.637931	-0.011353
$Pt(PMe_3)_2$ - 6	-1196.740685	0.257403	-8.624394	-0.021841
$Pt(PMe_3)(NH_3)$ - 1	-636.701004	0.112430	-4.064717	-0.022564
$Pt(PMe_3)(NH_3)$ - 2	-3288.377387	0.149152	-5.487242	-0.037930
$Pt(PMe_3)(NH_3)$ - 3	-3288.381047	0.149568	-5.498258	-0.030977

$Pt(PMe_3)(NH_3)$ - 4	-947.310474	0.266366	-8.749412	-0.026894
$Pt(PMe_3)(NH_3)$ - 5	-792.422025	0.188764	-6.579642	-0.024213
$Pt(PMe_3)(NH_3)$ - 6	-792.426334	0.188808	-6.572109	-0.033225
$Cu(CO)_2$ - 1	-1866.471921	-0.013409	-1.283602	-0.100698
$Cu(CO)_2$ - 2	--	--	--	--
$Cu(CO)_2$ - 3	--	--	--	--
$Cu(CO)_2$ - 4	-2176.968314	0.136785	-5.869175	-0.088082
$Cu(CO)_2$ - 5	--	--	--	--
$Cu(CO)_2$ - 6	--	--	--	--
$Cu(NH_3)_2$ - 1	-1753.145743	0.047602	-1.697199	-0.116210
$Cu(NH_3)_2$ - 2	-4404.726048	0.081805	-3.017533	-0.130643
$Cu(NH_3)_2$ - 3	-4404.719755	0.082526	-3.008896	-0.139908
$Cu(NH_3)_2$ - 4	-2063.670888	0.201210	-6.290567	-0.124645
$Cu(NH_3)_2$ - 5	-1908.773122	0.123282	-4.106435	-0.125477
$Cu(NH_3)_2$ - 6	--	--	--	--
$Cu(acetone)_2$ - 1	-2025.926580	0.127969	-4.927473	-0.077137
$Cu(acetone)_2$ - 2	--	--	--	--
$Cu(acetone)_2$ - 3	-4677.493638	0.165755	-6.236718	-0.087711
$Cu(acetone)_2$ - 4	-2336.445181	0.282654	-9.520009	-0.078990
$Cu(acetone)_2$ - 5	-2181.545890	0.207679	-7.333759	-0.079108
$Cu(acetone)_2$ - 6	--	--	--	--
$Cu(carbene)_2$ - 1	-2408.396421	0.357059	-11.678040	-0.049226
$Cu(carbene)_2$ - 2	--	--	--	--
$Cu(carbene)_2$ - 3	--	--	--	--
$Cu(carbene)_2$ - 4	-2718.944003	0.515188	-16.276189	-0.074539
$Cu(carbene)_2$ - 5	-2564.055075	0.434781	-14.102135	-0.072516
$Cu(carbene)_2$ - 6	-2564.052155	0.435265	-14.095349	-0.075177
$Cu(PMe_3)_2$ - 1	-2561.752480	0.180468	-5.781556	-0.073941
$Cu(PMe_3)_2$ - 2	--	--	--	--
$Cu(PMe_3)_2$ - 3	-5213.364999	0.217828	-7.136858	-0.075465
$Cu(PMe_3)_2$ - 4	-2872.302171	0.339650	-10.392518	-0.076899
$Cu(PMe_3)_2$ - 5	-2717.417427	0.257249	-8.227213	-0.074097
$Cu(PMe_3)_2$ - 6	--	--	--	--
$Cu(PMe_3)(NH_3)$ - 1	-2157.450267	0.114068	-3.741065	-0.094036
$Cu(PMe_3)(NH_3)$ - 2	--	--	--	--
$Cu(PMe_3)(NH_3)$ - 3	--	--	--	--
$Cu(PMe_3)(NH_3)$ - 4	-2467.994821	0.270519	-8.350371	-0.094834
$Cu(PMe_3)(NH_3)$ - 5	-2313.102539	0.192225	-6.176603	-0.092179
$Cu(PMe_3)(NH_3)$ - 6	--	--	--	--
$Ag(CO)_2$ - 1	-373.201492	-0.014858	-1.251676	-0.096649
$Ag(CO)_2$ - 2	--	--	--	--
$Ag(CO)_2$ - 3	-3024.737123	0.016311	-2.533491	-0.100566
$Ag(CO)_2$ - 4	-683.681065	0.134767	-5.808207	-0.089029
$Ag(CO)_2$ - 5	-528.796983	0.055989	-3.646104	-0.086385
$Ag(CO)_2$ - 6	--	--	--	--
$Ag(NH_3)_2$ - 1	-259.878355	0.046635	-1.658663	-0.110473
$Ag(NH_3)_2$ - 2	-2911.446858	0.078761	-2.963038	-0.132239
$Ag(NH_3)_2$ - 3	-2911.440900	0.080867	-2.955109	-0.140822
$Ag(NH_3)_2$ - 4	-570.381660	0.199792	-6.226240	-0.126676
$Ag(NH_3)_2$ - 5	-415.487058	0.120986	-4.049949	-0.124585
$Ag(NH_3)_2$ - 6	--	--	--	--
$Ag(acetone)_2$ - 1	-532.662913	0.125020	-4.889460	-0.076723
$Ag(acetone)_2$ - 2	--	--	--	--
$Ag(acetone)_2$ - 3	-3184.213697	0.159853	-6.178802	-0.087916
$Ag(acetone)_2$ - 4	-843.156575	0.279079	-9.453901	-0.078829
$Ag(acetone)_2$ - 5	-688.260487	0.203152	-7.270213	-0.078853
$Ag(acetone)_2$ - 6	--	--	--	--
$Ag(carbene)_2$ - 1	-915.129530	0.357420	-11.638306	-0.048341
$Ag(carbene)_2$ - 2	-3566.711729	0.395591	-12.933939	-0.085008
$Ag(carbene)_2$ - 3	-3566.719445	0.392554	-12.946571	-0.080269

$Ag(carbene)_2$ - 4	-1225.654929	0.510563	-16.209458	-0.074996
$Ag(carbene)_2$ - 5	-1070.764639	0.430733	-14.034656	-0.073171
$Ag(carbene)_2$ - 6	-1070.765665	0.435588	-14.034910	-0.076793
$Ag(PMe_3)_2$ - 1	-1068.497570	0.181242	-5.754340	-0.072603
$Ag(PMe_3)_2$ - 2	-3720.079508	0.217171	-7.062979	-0.088146
$Ag(PMe_3)_2$ - 3	-3720.091134	0.218321	-7.086844	-0.076734
$Ag(PMe_3)_2$ - 4	-1379.021933	0.337001	-10.337017	-0.077990
$Ag(PMe_3)_2$ - 5	-1224.136357	0.255857	-8.174014	-0.073744
$Ag(PMe_3)_2$ - 6	-1224.137123	0.255290	-8.170738	-0.075995
$Ag(PMe_3)(NH_3)$ - 1	-664.189561	0.112343	-3.709255	-0.089859
$Ag(PMe_3)(NH_3)$ - 2	-3315.771474	0.148898	-5.019771	-0.110235
$Ag(PMe_3)(NH_3)$ - 3	-3315.772271	0.148414	-5.027905	-0.103037
$Ag(PMe_3)(NH_3)$ - 4	-974.708417	0.267398	-8.290512	-0.095767
$Ag(PMe_3)(NH_3)$ - 5	-819.818198	0.187819	-6.120673	-0.091049
$Ag(PMe_3)(NH_3)$ - 6	-819.826180	0.186497	-6.124630	-0.093782
$Au(CO)_2$ - 1	-361.963695	-0.013826	-1.268662	-0.094569
$Au(CO)_2$ - 2	-3013.530273	0.016393	-2.594606	-0.092053
$Au(CO)_2$ - 3	-3013.518207	0.016604	-2.585808	-0.095934
$Au(CO)_2$ - 4	-672.465539	0.136872	-5.858246	-0.087268
$Au(CO)_2$ - 5	-517.588866	0.056193	-3.700968	-0.086163
$Au(CO)_2$ - 6	-517.622348	0.056937	-3.728024	-0.084803
$Au(NH_3)_2$ - 1	-248.638931	0.047432	-1.670260	-0.123204
$Au(NH_3)_2$ - 2	-2900.230275	0.081915	-3.005403	-0.141846
$Au(NH_3)_2$ - 3	-2900.228399	0.081286	-3.004296	-0.146721
$Au(NH_3)_2$ - 4	-559.172765	0.201966	-6.274999	-0.134601
$Au(NH_3)_2$ - 5	-404.282751	0.123410	-4.102963	-0.134146
$Au(NH_3)_2$ - 6	-404.311871	0.121917	-4.133522	-0.123919
$Au(acetone)_2$ - 1	-521.403113	0.127668	-4.885180	-0.074318
$Au(acetone)_2$ - 2	-3173.008470	0.161607	-6.239105	-0.081822
$Au(acetone)_2$ - 3	-3172.986302	0.162238	-6.217335	-0.086412
$Au(acetone)_2$ - 4	-831.932230	0.282764	-9.492476	-0.079076
$Au(acetone)_2$ - 5	-677.041737	0.205708	-7.314776	-0.080455
$Au(acetone)_2$ - 6	-677.097643	0.202356	-7.370489	-0.076692
$Au(carbene)_2$ - 1	-903.912058	0.358816	-11.676186	-0.053291
$Au(carbene)_2$ - 2	-3555.510172	0.400520	-12.999479	-0.084195
$Au(carbene)_2$ - 3	-3555.517866	0.394798	-13.013886	-0.078436
$Au(carbene)_2$ - 4	-1214.458075	0.516655	-16.278320	-0.074342
$Au(carbene)_2$ - 5	-1059.571567	0.432135	-14.110417	-0.072613
$Au(carbene)_2$ - 6	-1059.573672	0.438070	-14.109216	-0.077140
$Au(PMe_3)_2$ - 1	-1057.274077	0.178983	-5.793283	-0.071763
$Au(PMe_3)_2$ - 2	-3708.869825	0.217289	-7.123183	-0.087076
$Au(PMe_3)_2$ - 3	-3708.882794	0.219709	-7.144018	-0.079483
$Au(PMe_3)_2$ - 4	-1367.815758	0.338397	-10.398745	-0.077110
$Au(PMe_3)_2$ - 5	-1212.936772	0.256923	-8.243595	-0.073671
$Au(PMe_3)_2$ - 6	-1212.936173	0.255147	-8.237214	-0.076877
$Au(PMe_3)(NH_3)$ - 1	-652.960188	0.112669	-3.737629	-0.093888
$Au(PMe_3)(NH_3)$ - 2	-3304.560072	0.149756	-5.074099	-0.111421
$Au(PMe_3)(NH_3)$ - 3	-3304.561819	0.150595	-5.083565	-0.104126
$Au(PMe_3)(NH_3)$ - 4	-963.499840	0.269798	-8.346913	-0.097065
$Au(PMe_3)(NH_3)$ - 5	-808.616398	0.189132	-6.183584	-0.093939
$Au(PMe_3)(NH_3)$ - 6	-808.624030	0.190056	-6.184229	-0.099936

Table S3: Electronic energies, free energy corrections, and solvation corrrections of relevant species. Values in hartree.
